# The Role of the Japanese Traditional Diet in Healthy and Sustainable Dietary Patterns around the World

**DOI:** 10.3390/nu10020173

**Published:** 2018-02-03

**Authors:** Ana San Gabriel, Kumiko Ninomiya, Hisayuki Uneyama

**Affiliations:** Science Group, Global Communications Department, Ajinomoto Co., Inc., 15-1, Kyobashi 1-Chome, Chuo-ku, Tokyo 104-8315, Japan; kumiko_ninomiya@ajinomoto.com (K.N.); hisayuki_uneyama@ajinomoto.com (H.U.)

**Keywords:** healthy dietary patterns, Washoku, umami, glutamate, taste, Japanese cuisine, traditional diets, vegetables, taste receptors, dietary guidelines

## Abstract

As incomes steadily increase globally, traditional diets have been displaced by diets that are usually animal-based with a high content of “empty calories” or refined sugars, refined fats, and alcohol. Dietary transition coupled with the expansion of urbanization and lower physical activity have been linked to the global growth in the prevalence of obesity, overweight and life style-related non-communicable diseases. The challenge is in how to reverse the trend of high consumption of less healthy food by more healthful and more environmentally sustainable diets. The increasing recognition that each individual has specific needs depending on age, metabolic condition, and genetic profile adds complexity to general nutritional considerations. If we were to promote the consumption of low-energy and low salt but nutritious diets, taste becomes a relevant food quality. The Japanese traditional diet (Washoku), which is characterized by high consumption of fish and soybean products and low consumption of animal fat and meat, relies on the effective use of umami taste to enhance palatability. There may be a link between Washoku and the longevity of the people in Japan. Thus Washoku and umami may be valuable tools to support healthy eating.

## 1. The Traditional Japanese Diet and Its Potential Health Benefits 

### 1.1. The Importance of Umami Taste in Foods and Its Application

Much has been written in the last twenty years about umami as the fifth basic taste, also known in English as the “savory” taste. Umami taste is elicited primarily by the free amino acid glutamate, which is commercially prepared as sodium salt, hence its shortened name, MSG or monosodium glutamate. This savory taste characterizes many traditional Japanese foods. It is now believed that there are several identifiable receptor mechanisms responsible for detecting the taste of glutamate on the tongue and the palate [1,2,3]. 

Ikeda [4], who first identified glutamate as the primary umami taste compound, proposed that it served to identify sources of protein and consequently, some have proposed that protein status may be important for the sensitivity to umami. Early studies showed that both, well-nourished and malnourished infants preferred a soup with the seasoning MSG [5]. However, recently, Masic and Yeomans analyzed the liking for umami among high and low protein consumers and they found that the liking for MSG was rated as more pleasant when high protein consumers were in protein deficit [6]. More work is needed to understand the relationship between umami sensation preference and nutritional needs. Interestingly, even though no link has been found between the perception of umami taste with specific health outcomes, Pepino and colleagues [7] reported a lower sensitivity to MSG among obese women who preferred higher levels of MSG compared to normal-weight women.

Thanks to the extensive analysis in food ingredients of the levels of glutamate and two of the most abundant 5′-ribonucleotides, inosine monophosphate (IMP) and guanosine monophosphate (GMP), which synergize with glutamate to increase umami taste in foods, food technologists have identified foods that are naturally rich in umami substances, such as soup stocks, mushrooms, tomatoes, and fermented cheeses [8]. However, the characteristics of umami taste in complex food systems need to be studied in more detail. Thus, the authors here will focus on the evidence that explains the unique role that umami plays in the Japanese traditional diet, known as Washoku. We also discuss its potential application in other diets. 

The Japanese soup stock *dashi* contains a significant amount of glutamate and IMP or GMP, depending of the type of *dashi*. It is believed that the particular profile of umami substances in *dashi* enhances the original flavors of foods and increases their palatability [9,10]. The effect of umami substances is described as “meaty and mouthful”, “coating sensation” or even tactile. How can umami compounds exert this function in foods? From a food technology and physiological point of view, the exact mechanism by which glutamate and 5′-ribonucleotides function to create this effect cannot be fully explained by the activation of glutamate receptors on the tongue. 

Glutamate plays an important role in the palatability of foods, and its palatability is not entirely due to learning. Early behavioral studies based on the analysis of facial expressions in neonates showed that the addition of 0.5% MSG was able to reverse the typical aversive response of spitting and gaping to a clear vegetable soup. In fact, newborn infants displayed a similar response to soup with added MSG as they do to sweet solutions: sucking and positive facial expressions [11]. This reaction of acceptance of MSG in soups by newborns is representative of the effect of glutamate in other foods in adults as well as children. Strangely, in an aqueous solution, MSG is unpalatable to both adults and infants. The reason for this is obscure [12]. In short, the optimal concentration of MSG, which usually ranges from 0.04% to 1.6%, has the ability to increase the acceptability of foods by changing the sensory and consequently, hedonic or pleasant properties of food. 

Added glutamate also increases the liking of novel flavors, in much the same way that fat and sugar do [13]. Sugar and fat are thought to influence liking via their caloric content and reward effect. It is not clear in the case of MSG how umami influences liking. The increase in palatability by MSG is so robust that it can maintain the acceptability of food with reduced salt, which also works by improving the perception and flavor intensity in food [14,15,16,17,18]. That is, studies have confirmed that the partial substitution of salt by MSG allows for an overall decrease in sodium without reducing food palatability. Thus, added MSG could be an effective strategy to decrease sodium concentration in foods. Prescott and Young [19] illustrated how MSG increases the acceptability of soups, even among consumers that have a negative outlook towards MSG. Consumers rated the flavor of foods with added MSG as significantly better liked, richer, saltier, and more natural tasting. This higher food acceptability after adding MSG also influences food choices and, consequently, food intake. This property has been used to improve the nutritional status of older individuals [20,21]. Altogether, substantial research indicates that MSG and natural glutamates from *dashi* or other foods rich in umami could play a role in enhancing the palatability and promoting the consumption of nutritious foods with low sodium content. It thus has the potential to be strategically used to decrease the intake of animal-based ingredients and enhance intake of others that promote overall health, such as vegetables, as is done in Washoku. There is a long history for the use of MSG as a flavor enhancer, which the Food and Drug Administration of the United States has categorize as generally recognized as safe (GRAS) [22,23]. 

### 1.2. How Does Umami Enhances the Palatability of Foods?

The answer to this question is still unclear but there are several possible explanations. Part of the effect of MSG in foods could be explained by the content of sodium in MSG. However, Okiyama and Beauchamp [24] found that when comparing two soups with the same amount of sodium, subjects still preferred the one with MSG. The interaction of umami with other tastes modalities could be another reason. This interaction can work in two ways, either on taste intensity or on the temporal evolution of a taste sensation, also known as temporal dominance of sensation (TDS) [25,26]. In regard to taste intensity, umami sensation can enhance the perception of saltiness and make sourness more pleasant. There is also some evidence to suggest that glutamate can augment the perception of sweetness and suppress the intensity of some bitter compounds [25]. Recently, umami taste interaction with salty and sour tastes have also have been analyzed from a temporal point of view [26]. One study has shown that when MSG is combined with either NaCl (salty taste) or lactic acid (sour taste) the duration of the umami sensation was altered. IMP and NaCl decrease the duration of umami taste, whereas MSG suppresses the duration of the sourness of lactic acid. 

Umami sensation increases salivary secretion, and this increase over 10 min is larger than that elicited by sour stimuli [27,28]. This property may be another way for glutamate to enhance food palatability. Saliva serves as a vehicle to dissolve the taste substances from foods and protect the proper functioning of taste sensation [29]. Hyposalivation can alter taste perception, which may result in poor appetite, weight loss and poor general health. Umami taste stimulation has been employed therapeutically to improve the flow of salivary secretion in elderly patients who have deficient umami taste sensation [30]. 

Another important physiological function of glutamate worth mentioning is its role as a signaling molecule in the gastrointestinal tract. Glutamate receptors have been found in the stomach and the gut [31,32], and studies suggest that glutamate may enhance food signaling to the brain by stimulating the vagus nerve and the secretion of neuroendocrine hormones and digestive juices that support the digestion of proteins [33,34]. 

And lastly, recently, it has been found that the umami sensation interacts with odors, as sweet and sour tastes do, by enhancing the intensity of aromas, such as that of chicken soup or celery (phthalide compounds), especially when these foods are swallowed [35]. Altogether, in addition to the modality of ‘mouth feel’ of umami that influences the body and thickness of a dish, it seems that glutamate enhances appetitive sensorial traits in a complex food context while masking the negative ones. At the same time umami is involved in the regulation of various gastrointestinal functions (review, [36]). This could partially explain why there is no need in Japanese traditional diets to use large amounts of animal fat or meats for optimal palatability—the meat-like sensation of traditional Japanese dishes with umami is sufficient. 

### 1.3. The Traditional Japanese Cuisine, Washoku: Why Is It Thought to Be Healthy?

**Figure 1:**
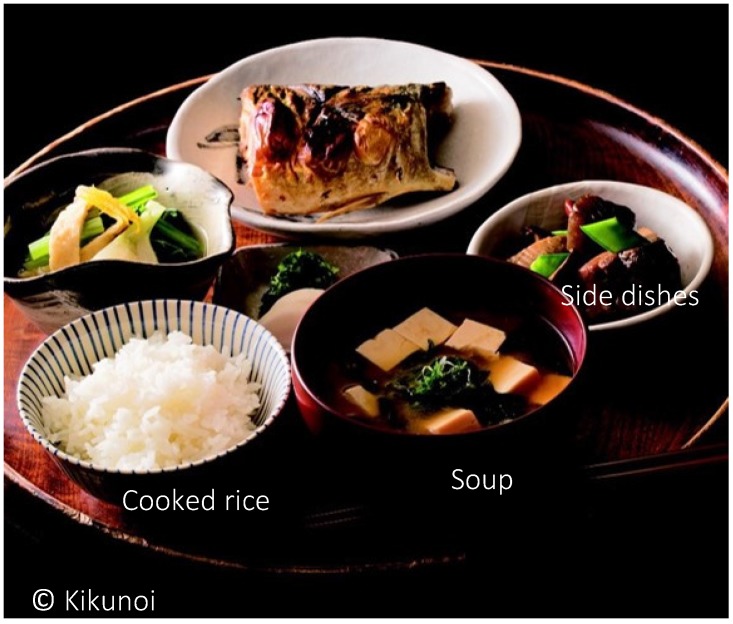
The basic structure of Washoku, comprised of one soup, cooked rice, and three side dishes, deliciously prepared with *dashi* stock as accompaniment for the rice.

The traditional dietary cultures of Japan are collectively known as Washoku. In 2013, Washoku was named in the UNESCO list of Intangible Cultural Heritage. According to Professor Kumakura Isao, the President of the National Assembly on the Preservation and Continuation of Washoku culture, the guiding principles of Washoku are a staple food—rice—which is complemented by a variety of side dishes, soup, and pickles. Together these form the basic structure of a meal, customarily eaten using chopsticks, wooden bowls known as “wan”, and the like (Figure 1, Table 1). This menu benefits fully from the distinctive flavor (combination of taste, smell, and tactile sensations) of each ingredient.

This style of eating a main staple food with side dishes interchangeably, is unique to Washoku, mixes, and harmonizes all flavors inside the mouth. Small bites, due to the use of chopsticks, together with the combination of foods inside the mouth seem to contribute to satiety. There is evidence showing that multiple alternation of foods decreases food consumption at the end of the meal [37]. The relatively small portion size of the main and side dishes is another trait that helps to avoid overeating, since studies have shown that big portions encourage the consumption of larger meals [38,39]. Frequent intake of soup by Japanese men has been correlated with a lower body mass index (BMI), waist circumference, and waist-to-hip ratio, all physical factors related to obesity [40]. Others have also demonstrated that soups have a satiating effect [41,42]. In fact, the core flavor of Japanese food is umami taste from *dashi* stock, which is the base of many Japanese recipes. To heighten the distinctive flavor of many ingredients, cooks in Japan have mastered the techniques of extracting umami substances from dried kelp and dried bonito flakes in *dashi* stock with traditional flavoring products, such as soy sauce, miso, and vinegar [9]. 

Water is another important ingredient in traditional diets. As rivers in Japan are short, water is soft and quite free of impurities. Thanks to the work of culinary professionals at the Japanese Culinary Academy, it is known that soft water functions not only to reduce or remove bitterness but it also efficiently brings out the umami sensation from dried kelp and dried bonito flakes. This *dashi* stock is used to boil vegetables and serves two functions: It reduces the volume and increases the palatability of vegetables. This facilitates the inclusion of larger quantities of vegetables within the Japanese menu and thereby increases their consumption, which has been shown to lead to a lower the risk of cardiovascular diseases (CVD) and all causes of mortality and morbidity [43]. Moreover, the main cooking methods in Washoku are steaming, boiling, and stewing, thereby enhancing the water content of Japanese dishes. This incorporation of water into food seems to be more efficient that drinking water to decrease the overall intake of energy in a meal [44]. 

Altogether, the style of eating in Washoku—a large variety of foods, small portions, the inclusion of soups, abundant vegetables, the cooking method, the large content of water, and the effective usage of umami taste—promotes not only the pleasant experience of eating, combined with the large incorporation of bioactive compounds from vegetables, but also ensures an adequate signal for satiety that prevents overeating. Another parameter to take into account as a potential healthful trait of the Japanese diet is the frequent consumption of fish. Side dishes in Washoku include many types of fish that are a rich source of high quality protein as well as eicosapentaenoic acid (EPA) and docosahexaenoic acid (DHA), ω-3 fatty acids that are believed to be beneficial for health [45]. Soy bean-based foods, in the form of fermented miso and tofu, are common in Japanese traditional diets, and are known to reduce blood pressure and blood glucose [46,47]. Additional factors to consider are the energy and sodium content of the Japanese diet. Several studies have found consistent low calorie ingestion among men and women from Japan, compared to those in China, the United States, Italy or the UK [48,49]. This may partly explain the lower BMI among Japanese compared to other populations. In reference to sodium, a high urinary excretion has been reported for Japanese people, accompanied by a high estimated sodium consumption—between 11 mg for men and 9 mg for women daily. Although salt intake in Japan, especially in certain regions, has considerably decreased from the 1950s and 1960s, the current consumption is still higher than the recommended amount to reduce mortality by stroke (<6 mg per day) [50,51]. The most common dietary sources of sodium in the Japanese diet are miso soup and salted vegetables as well as soy sauce and commercially processed fish or seafood. However, in spite of a high sodium intake, Japanese have an overall low incidence of CVD, probably due to a higher potassium intake with vegetables [52]. Finally, families strengthen their bonds by sharing meals together, which is important for usual communication [53]. In summary, the main elements of Washoku that promote positive health outcomes are: (1) the great variety of seasonal foodstuffs, including vegetables and fishes; (2) the way of cooking dishes based on large amounts of high quality water; (3) the well-balanced nutrition; and finally (4) the value of its connection with health and family ties (Table 2 and Table 3) [9].

## 2. Food Polyphenols and Their Sensory Properties 

The sensorial properties of foods depend basically on two major factors: the amount and type of taste active compounds from foods and the distinct sensitivity and taste experience of each individual. Flavonoid phenols, such as flavonols, which are present in several fruits, nuts, chocolate and beverages like tea, cider, and red wine, provide a characteristic bitterness and the tactile feeling of astringency (puckering, rough or drying mouth-feel). Although astringent molecules may have protective effects in our body, excess astringency can be unpalatable. These sensorial properties are best known to arise from the monomers of flavan-3-ol that are called proanthocyanidins, and also condensed tannins (epigallocatechin, epicatechin gallate, epigallocatechin gallate, catechin and epicatechin) [54]. They can be found in wine and tea and are thought to be responsible for the bitterness and astringency of both drinks. Small changes in the chemical structure of flavonoids can induce significant differences in sensory properties. For example, catechin is less bitter and astringent than its chiral isomer, epicatechin. On the other hand, bitterness declines, whereas astringency intensifies, with increasing polymerization of flavonoids. Moreover, the interactions between flavonoids and other food compounds, such as ethanol, can enhance the intensity of bitterness in wine without affecting its astringency. Oral sensory perception of astringency comes, in part, from the reduction of oral lubrication after the precipitation of certain salivary proline-rich proteins that show a strong binding affinity for polyphenols, like tannins [55]. However, astringency involves an intricate mechanism that gives a complex sensation that is not yet fully understood [56,57]. 

The sensorial properties of other phenolic compounds have also been studied. Over the past ten years, the peculiar pharyngeal pungency of a phenolic constituent present in newly pressed extra virgin olive oils (EVOOs), known as oleocanthal (OC) [(−)-decarboxymethyl ligstroside aglycone], has been investigated by Beauchamp and colleagues [58]. OC appears to have very similar pharmacological activity to ibuprofen in the inflammatory pathways and also a similar oropharyngeal irritation. This makes OC a natural nonsteroidal anti-inflammatory compound and could partly explain the beneficial effects of the Mediterranean diet, together with protective effects of other antioxidant polyphenols of olive oil [59]. The sensory properties of OC seem to be mediated by a type of transient receptor potential receptor (TRPA1) that has been shown to be involved in the transduction of pain, due to thermal, mechanical, and chemical signals [60,61].

## 3. Why Taste Matters? 

Taste matters for food selection and utilization, for several reasons. Firstly, taste is considered the nutrition gatekeeper, thereby helping individuals determine food acceptability, which is lifesaving for all animals, including humans. Most taste researchers believe that there are five basic or primary taste qualities: sweet, salty, sour, bitter, and umami (savory or meat-like). Sweet and umami taste perception probably functions to sense energetic sources, in particular, to recognize carbohydrates and proteins, respectively. A strong sour taste may serve to identify spoiled foods, while a salty taste acts to recognize the presence of sodium, which is necessary for the homeostasis of body fluids. Finally, strong bitter tastes aid in detecting the presence of toxicity [62,63,64], while a mild bitterness might potentially indicate the presence of medicinal compounds. 

Taste remains important in food selection, even in developed cultures. This is indicated by results of study of food-related values that individuals use to make food choices. According to the Food Choice Process Model, these values include health, cost, time and social relationships. Of these, taste is among the top motives [65]. Thus, it is expected that taste perception and preference significantly influence food intake behaviors [66,67,68]. Taste perception varies among individuals due to genetic variations of taste receptors that can lead to adverse eating behaviors among some individuals and consequently, to a greater risk of chronic diseases [64,69]. This is discussed in greater detail below. On the other hand, some researchers think that it is necessary to have prolonged oro-sensory exposures to taste for a sufficient cephalic phase and satiety responses [70]. This would be the reason why slow eating elicits a robust satiety signal, whereas energy-containing beverages are only briefly tasted and thus provide a weak satiety signal. 

### 3.1. Genetic Variation of Taste Receptors: Bitter Taste

Bitter taste perception is a consequence of certain molecules interacting with bitter taste receptors, called *TAS2Rs*. These receptors are a group of 25 G protein-coupled receptors that transduce the bitter taste sensation [71] The most commonly researched genetic taste variation is the inherited polymorphism of one these bitter taste receptors, *TAS2R38*. By chance, a chemist at DuPont laboratories (Arthus Fox) found in 1932 that some people could not detect certain bitter compounds, whereas others found them extremely bitter [72]. Later, Linda Bartoshuk studied the genetic differences of bitter taste perception in more detail [73]. She found that 25% of the mainly Caucasian population she examined was particularly sensitive to a group of bitter organosulfur thiourea compounds—phenylthiocarbamide (PTC) and 6-n-propylthiouracil (PROP). They contain a phenyl ring and are considered to be potent disruptors of several enzymes produced by the thyroid gland (goitrogens). Although neither PTC nor PROP is present in foods, cruciferous vegetables such as cabbage, broccoli and brussel sprouts contain chemically related glucosinolates with distinctive thiourea moieties [74]. Those who could not taste PTC or PROP, except at very high concentrations, accounted for approximately 25% to 30% of this population and were termed non-tasters. In marked contrast, approximately 25% of this population was extremely sensitive to these compounds. This extremely sensitive group she called “super-tasters”. The remaining 45 to 50% of the population was average in their ability to taste PROP. 

The major factor explaining individual variation in bitter taste perception of these compounds is genetic polymorphism at the taste receptor level, or at the messenger RNA expression level of the receptor. There are three major polymorphisms of the *TAS2R38* gene that are responsible for the perception of PROP, PTC, and other thiourea related compounds. These combine to form most of the taster haplotypes. However, changes in the thresholds of perception may also be related to the differences in the expression of *TAS2R38* [75]. 

Previous surveys have already noted that individuals who have a higher taste sensitivity to PTC or PROP are prone to disliking pungent foods with strong tasting qualities, compared to non-tasters who, having lower taste sensitivity, experience more pleasant taste sensations from foods in general. Some studies have shown that there seems to be a link between the sensitivity to PROP, food perception, food preference, and finally food choice, which could potentially predict the risk of chronic diseases [66,74]. As attractive as this hypothesis linking differences in genetic sensitivity to food choice might be, taste sensitivity, or PROP genotype, are not the only aspects that influence food choice or dietary intake. Other factors, such as culture or experience, education, socioeconomic status, peers, individual characteristics in relation to health, sex, age and body weight, also influence the food we prefer to eat [74]. 

### 3.2. Other Taste Receptor Variants and Taste Perception

Single nucleotide polymorphisms (SNPs) have been also found in other taste genes. Among these are potential fatty acid taste receptor cluster determinant 36 (CD36), the umami heterodimer taste receptors, type 1 member 1 (T1R1) and T1R3, the heterodimer sweet taste receptors (T1R2 & T1R3), the salt taste epithelial sodium channel (ENaC), and the transient receptor potential cation channels (TRPV1) (reviews: [1,64]). Although fat taste (oleogustus), the taste of triacylgrycerols, as a basic taste, is still in dispute, energy-dense foods that are high in fat may contribute to a higher palatability and predispose individuals to metabolic diseases [64]. This palatability could be different from the sensation of non-esterified, long chain fatty acids (NEFA), which are part of fatty foods in small amounts. Evidence points to the possibility that humans taste NEFA as a unique sensation [76]. A substantial individual variability has been reported for the sensitivity of NEFA in humans [77,78]. Some studies have found that a lower sensitivity to NEFA perception is linked to a higher energy and fat consumption, and consequently, a higher BMI [79,80,81]. Differences in sensitivity could be in part due to SNPs or the expression level of CD36 that is involved not only in taste but also in lipid metabolism and the risk of CVD [80,82]. Low sensitivity for the taste associated with CD36 seems to promote fat intake, which would explain, in part, why obese individuals eat fatty foods more often [68]. However, more studies are necessary for a better understanding of differences in taste sensitivity between lean and obese individuals [83]. 

For the sweet taste receptor, *TAS1R2* seems to be the human gene with one of the highest polymorphic rates [84]. Most of the SNPs seem to be located at the sequence where ligands bind to the receptor. Some studies have associated T1R2 and T1R3 receptor variations with taste sensitivity to sweet and sugar intake, obesity and dental caries [85,86,87]. 

Umami taste is represented most prominently by the taste of monosodium glutamate (MSG) and by its synergistic interaction with the 5′ribonucleotides: inosine monophosphate (IMP), guanosine monophosphate (GMP), and adenosine monophosphate (AMP). For MSG, there are also studies that show individual differences in sensitivity [88]. Some of these differences may come partly from SNPs in T1R1 and T1R3 receptors [89].

In contrast to the lack of studies showing a link between umami taste and diseases, excess sodium intake presents a major public health concern because of its relationship with the development of high blood pressure. As indicated before, supertasters report a stronger perception of the saltiness of concentrated salt solutions than do non-tasters [67]. However, sensory habituation to high dietary sodium appears to play a greater role in defining inter-individual differences for salt preference [90]. Unfortunately, salt taste receptors are not sufficiently characterized to draw conclusive implications on their role in behavioral preference to sodium and health effects. Currently, it is accepted that there are two responses to sodium. The appealing taste sensation of low-to-moderate sodium concentration seems to be mediated by the protein ENaC, whereas the TRPV1 system may be more related to aversive reactions to the taste of very high concentrations of sodium [91]. Polymorphisms related to the perception of salty taste intensity have been reported for both ENaC and TRVP1 [92]. Further research is needed to understand the extent to which the SNPs of salt taste receptors are involved in the preference for salty foods. 

Lastly, coding SNPs have been also described in the genes of the presumed sour taste receptors, PKD2L1 and PKD1L3. They belong to a subfamily of transient receptor potential ion channels—polycystic kidney diseases-like (PKDL). However, the effect of these SNPs in the perception of sour taste is not yet well known (for review [64]). Altogether, current research shows that particular genetic variations of the fat, salty, sweet, and bitter taste receptors may predispose individuals to eat less vegetables (healthy foods) and overconsume fat, salt, and sugar (unhealthy foods), Table 4. 

## 4. Is the Current Diet of the Japanese People Healthy? 

Japan is among the nations with the highest average life span for both, men and women, a fact consistent with the potential benefits of the traditional Japanese diet [93,94,95]. The culture of the traditional diet has been broadly maintained with a high intake of fish and soybean products and low intake of fat. At the same time, it has been also characterized by a high salt consumption [96]. However, in spite of a higher sodium intake and prevalence of high blood pressure, Japan still has lower mortality rates caused by CVD than Western nations [97]. For cultural and religious reasons, the Japanese have traditionally avoided the use of animal meats. During the Japanese economic development and the dramatic surge in the variety of available ingredients, the nutritional balance improved considerably in the 1980s, which, for most Japanese, reached an almost ideal balance of protein, fat, and carbohydrates. 

In 2005, the Japanese Ministry of Health, Labour and Welfare developed, jointly with the Ministry of Agriculture, Forestry and Fisheries, the Japanese Food Guide Spinning Top, based on the dietary guidelines for Japanese that were formulated in 2000 [98]. However, more recently, the incidence rates of obesity and the metabolic syndrome have increased among middle-aged men. Coincidentally, the rate of underweight young women who want be thin has also increased, while child obesity in both boys and girls is starting to be of concern [99]. In the last forty years, there has been a partial loss in traditional food culture among the Japanese population. They have taken up less healthy dietary habits, such as skipping breakfast, insufficient vegetable intake and excess fat intake, combined with an increase in consumption of meat, eggs, milk, and dairy products.

Following the recent inclusion of “Washoku, traditional dietary cultures of the Japanese” in the list of Intangible Cultural Heritage of UNESCO, the interest in the traditional Japanese diet has increased, with a renewed appreciation for its potential health benefits. This seems to be a positive result, since recent Japanese cohort studies have shown that individuals with greater adherence to the Spinning Top of the Japanese Food Guide have a lower total mortality rate of 15% in both men and women, mainly due to a reduction in cerebrovascular diseases [93]. Others have now created a modified score to measure diet quality for Japanese that is also based on the Japanese food guide Spinning Top, but includes intake of sodium from seasonings, which was not part of previous scores [100]. 

At the first World Food Summit, held in Rome in 1996, under the auspices of the Food and Agriculture Organization of the United Nations [101], it was acknowledged that the eating habits of Japanese people are unique, compared to those of other nations or regions. Moreover, “Eating deliciously” is a priority of Japanese citizens, according to a 2006 survey by the NHK Broadcasting Research Center [102]. This is facilitated by the wide use of umami rich *dashi* in traditional cuisine. Now, more studies on dietary health scores for Japanese are necessary, to develop specific strategies for improving the dietary habits of younger generations. However, they are also necessary because some of the concepts within the Japanese diet can be useful in increasing healthy dietary habits in other countries. 

## 5. Sustainability of Healthy Diets 

The Food and Agriculture Organization of the United Nations (FAO) has defined sustainable diets as those having “low environmental impacts that contribute to food and nutrition security.” Sustainable diets are also considered not only culturally acceptable, accessible and affordable, but also able to optimize natural and human resources [103]. The question is whether diets assessed as healthy can be also sustainable, because healthier diets are not necessarily more beneficial for the environment. Many recent studies looking at nutrition indicators, such as energy adequacy, food quality and composition, also address the environmental impact of a diet. [104]. Most of these studies refer to the Mediterranean diet. In general terms, plant-based foods produce fewer emissions of greenhouse gasses than animal-based foods. Sustainable vegetarian diets consist of grains, vegetables and fruits, with few servings of meat or seafood [105]. These are common ingredients in most traditional diets, including the Japanese traditional diet, and it will be necessary in the future to evaluate the impact that any diet may have on the environment in the region where it is implemented. 

## 6. Summary and Conclusions

**Figure 2:**
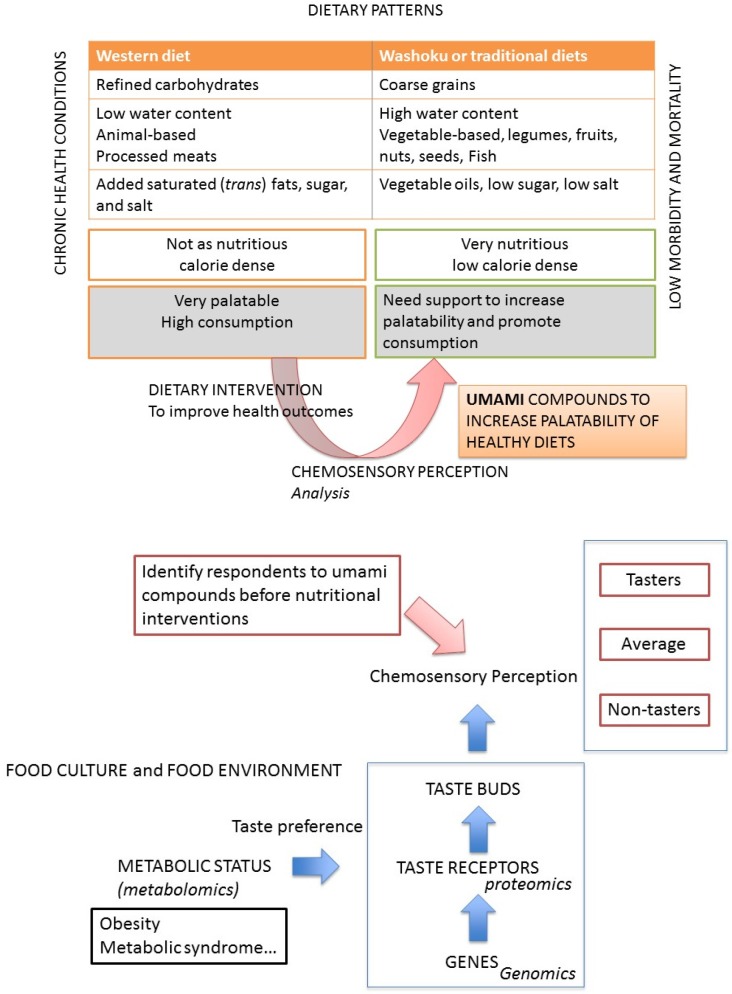
Traditional diets are usually associated with longevity and lower morbidity and mortality, but they are not as palatable as “Western diets”. Taking into account data on taste sensitivity in personalized nutrition, together with the better understanding of food consumption behavior, can ensure a better adherence to nutritional interventions.

In this article, we put forward an argument that Japanese traditional diet practices (Washoku), which prominently include the flavoring of foods with umami taste, can be characterized as a healthy diet in the same way that the DASH diet or the Mediterranean diet is so classified (summary in Figure 2). We then discussed the importance of taste in guiding food choice and the important role that genetically-based individual differences in taste perception can have on a person’s food selection behavior. We hope that several of the principles of Washoku will be studied and adopted by physicians, nutritionists, dieticians and others engaged in encouraging healthful eating. 

## Figures and Tables

**Table 1:** Characteristic dishes and ingredients of the Japanese traditional diet.

Dishes	Ingredients	Elements
Staple food	Grains, mainly rice (noodles or glutinous rice)	Recipes with cooked rice (sushi or curry rice)
Soup	Miso Soup (seaweed, shellfish, vegetables)	*Dashi* soup stock (fermented soybeans)
Main dish	Fish, seafood, sometimes meats	Great variety of edible fishes
Side dishes	Vegetables, wild plants, mushrooms, seaweed, shellfish	Change with the season and locality

**Table 2:** Basic elements of the Japanese traditional diet ^1^.

Elements	Contents	Description
Foodstuffs	Seasonal foods	Rice, vegetables, wild plants, mushrooms, variety of fish
Dishes	Cooking methods with abundant water, *dashi* stock, delicious meals, with vegetables and seafood	Steaming, boiling, and stewing
Nutrition	Relative low-calorie density, low total fat, high quality protein, variety of ingredients, easy to eat different nutrients	Nutritionally well-balanced
Hospitality	Health and family ties	The joy of eating together and caring for one another

^1^ The traditional Japanese diet starts with the selection of foodstuffs, and includes the way foods are prepared, how ingredients contribute to balanced nutrition, and finally, the attitude of appreciation.

**Table 3:** Potential health traits of Washoku.

Element	Effect	Health Consequences
Small portion size	Smaller meal size	Prevents overeating [38,39].
Soup and dishes with high water content	Lower total energy intake	Lower ^1^ BMI, waist circumference and waist-to-hip ratio [40,41,42,48,49].
Soy sauce, salted vegetables and fruits, miso soup, and salted fish	High sodium consumption, with a high sodium/potassium ratio	The high vegetable intake seems to protect against CVD [50,51,52].
High ^2^ EPA and ^3^ DHA, low animal fat, low total fat	Modulation of the membranes of cells, lipid signaling and gene expression	Supports optimal health, low risk of ^1,4^ CVD, cancer and inflammation [45]
Foods based on beans	Decrease blood pressure and blood glucose	Protects against CVD [46,47]
Variety of seasonal vegetables and green tea	Intestinal bulk and protection against inflammation and high blood pressure	Low risk of CVD and all causes of mortality [43].
Umami taste	Enhances flavor, food palatability and salivation	Promotes chewing and swallowing, and maintains adequate taste sensation [27,28,30].

^1^ Body Mass Index [BMI]; ^2^ Eicosapentaenoic acid (EPA); ^3^ Docosahexaenoic acid (DHA); ^4^ Cardiovascular diseases (CVD).

**Table 4:** Taste receptor genes with single nucleotide polymorphisms (SNPs) and their corresponding taste qualities.

Taste Quality	Taste Receptors with SNPs	Citation
Bitter	*TAS2R38*	[71]
Fatty Acids	*CD36*	[77,82]
Sweet	*TAS1R2/TAS1R3*	[84]
Umami	*TAS1R1/TAS1R3*	[89]
Salty	*ENaC*	[91,92]
Sour	*PKD2L1/PKD1L3*	[64]
